# Presence of the point mutations Val1016Gly in the voltage-gated sodium channel detected in a single mosquito from Panama

**DOI:** 10.1186/s13071-019-3309-y

**Published:** 2019-01-28

**Authors:** Osiris Murcia, Brigitte Henríquez, Angélica Castro, Susana Koo, Josue Young, Ricardo Márquez, Debora Pérez, Lorenzo Cáceres, Anayansi Valderrama

**Affiliations:** 1 Programa de Maestría en Ciencias Biológicas, Vicerrectoría de Investigación y Postgrado, Universidad de Panamá, Panamá, República de Panamá; 2 Universidad Autónoma de Chiriquí, Chiriquí, República de Panamá; 3 Department of Research in Medical Entomology, Gorgas Memorial Institute of Health Studies, Panama City, República de Panamá

**Keywords:** *kdr* mutations, Pyrethroid resistance, Voltage-gated sodium channel, *Aedes aegypti*, *Aedes albopictus*, Panama

## Abstract

**Background:**

In Panama, arboviroses such as dengue fever, and more recently chikungunya fever and Zika disease, are transmitted by *Aedes aegypti* and *Aedes albopictus*. Their control is based on the elimination of breeding sites and fogging with pyrethroid insecticides. However, one of the significant issues derived from the prolonged use of pyrethroid insecticide is the development of resistance mechanisms, such as knockdown resistance or *kdr*. The objective of this study was to evaluate the presence of *kdr* mutations in a partial region of the VGSC gene in samples of wild-caught *Aedes* mosquitoes from different locations of the Metropolitan Region of Panama.

**Results:**

Based on the analysis of 194 sequences of the VGSC gene, two *kdr* mutations (Ile1011Met and Val1016Gly) were detected in a specimen of *Ae. aegypti*. The frequency of *kdr* mutations in the evaluated samples of *Ae. aegypti* was 0.01.

**Conclusions:**

This study provides evidence for a low frequency of *kdr* mutations in *Ae. aegypti* populations in Panama. It is possible that these changes have no impact on vector control interventions. To our knowledge, we report, for the first time in America the Val1016Gly mutation documented in Asia. In general terms, this result is highly relevant to the *Aedes* Control Programme in Panama since it constitutes a feasible approach for the timely detection of resistance as well as for the development of strategies.

**Electronic supplementary material:**

The online version of this article (10.1186/s13071-019-3309-y) contains supplementary material, which is available to authorized users.

## Background

*Aedes aegypti* (Linnaeus) and *Aedes albopictus* (Skuse) are the two most important mosquito species in terms of the transmission of infectious diseases [1, 2]. They are vectors of different arboviruses (viruses transmitted by arthropods) of worldwide relevance that include dengue virus (DENV), yellow fever virus (YFV), chikungunya virus (CHIKV) and Zika virus (ZIKV) [3]. Both of these species of the order Diptera are considered invasive since they have shown success in colonizing many regions outside their endemic areas [2].

Currently, control activities are based on surveillance, chemical application and the elimination of mosquito breeding sites. The pyrethroid insecticides such as deltamethrin and cyfluthrin have been, until now, the most commonly used class of insecticide for vector control against the annual epidemics of diseases transmitted by *Aedes* in Panama [4, 5]. The rotation in the application of insecticides has also been carried out; deltamethrin has been applied to health regions that have the highest infestation rates and cyfluthrin in the remaining regions. However, even if outbreaks are controlled, concerns about the effect of the continuous use of these insecticides on the populations of *Ae*. *aegypti* and *Ae*. *albopictus* persist, specifically in relation to the development of resistance mechanisms, which is one of the main problems faced by chemical control programmes [5–7].

In Panama, populations of mosquito vectors have been subjected to a continuous selective pressure of organochlorine, carbamate, organophosphate and pyrethroid insecticides. The application of these chemical compounds began in 1957 through the creation of the National Service for the Eradication of Malaria (Servicio Nacional de Erradicación de la Malaria, SNEM) [8, 9]. However, activities for the control of *Aedes* populations officially began in the year 1969, through the Campaign of Eradication of *Ae. aegypti* developed by the Ministry of Health. During the 1980s, this was renamed Campaign for the Control of *Ae. aegypti* and, subsequently, MINSA integrated the SNEM with the Programme of *Ae. aegypti* and created the Department of Vector Control. Since 1969, temephos (Abate) has been used for the control of larvae (focal treatment) of *Aedes*. From the 1970s until 1985, malathion spraying was used as a perifocal treatment for the control of adult mosquito populations. From 1985 to the present, spraying with the pyrethroid deltamethrin has been applied [10]. From 1992 to 2016, spraying with cyfluthrin was applied in the regions with the lowest risk of transmission; however, its use was suspended due to supply problems.

Resistance is due to two main mechanisms: a greater metabolic detoxification of chemical agents and insensitivity at the site of action [11]. The mechanisms involving overexpression or qualitative changes in catalytic enzyme sites include non-specific esterases (NSE), glutathione S-transferases (GSTs) and mixed function oxidases (MFOs) [12]. However, knockdown resistance, or *kdr*, is one of the main types of resistance against pyrethroid insecticides [13]. It is caused by point mutations at the level of the nucleotide sequence of the *para* gene and leads to changes in some amino acids of the voltage-gated sodium channel (VGSC) protein, which causes a reduction of the binding with the insecticide and, consequently, the loss of its effect [14–17].

This study aimed to evaluate the resistance in *Aedes* vectors through molecular tools, given that previous studies with populations of *Ae*. *aegypti* have shown metabolic resistance, and its mechanisms have been characterized for different types of insecticides [18, 19]. Furthermore, there is no previous information about the presence of *kdr* alleles in the studied *Aedes* populations in Panama.

The study is part of a more comprehensive investigation that includes the performance of susceptibility bioassays standardized by the WHO, and the use of synergistic agents and biochemical tests for the detection of enzymatic mechanisms associated with resistance to insecticides. Each of these approaches satisfies the need of the *Aedes* Control Programme of MINSA to understand the behavior of resistance and its mechanisms in populations of these vectors.

## Methods

### Sampling

The sampling locations were selected based on the criteria of a high incidence of dengue and levels of infestation with *Aedes* mosquitoes from high to moderate [20]. Five locations of the Metropolitan Region of Panama were selected: Nuevo Chorrillo (8°57'36.09"N, 79°41'54.48"W), Princess Mía (8°58'1.29"N, 79°42'8.92"W), Lluvia de Oro (8°57'36.57"N, 79°41'56.28"W), Bethania (9°0'34.04"N, 79°31'45.95"W) and Las Garzas (9°7'6.00"N, 79°15'47.32"W). In each locality, 25–30 houses were selected at random.

The capture of *Aedes* mosquitoes and eggs was carried out during the months of August to November 2015. For this purpose, two trap types were used: a Mosquito Science Trap-N-Kill™ ovitrap (SpringStar Inc., Woodinville, WA, USA) and a a BG-Sentinel® Trap (Biogents AG, Regensburg, Germany), which were placed in the peridomicile of the homes. Within each BG-Sentinel Trap, BG-Lure™ (Biogents AG, Regensburg, Germany) was used as an attractant to favor the capture of adult mosquitoes. These traps were monitored daily to change the battery and the catch bag, the latter of which was transported to the laboratory in a portable ice cooler in order to preserve specimens for taxonomic identification and subsequent molecular analyses. In the case of the Trap-N-Kill™ ovitrap, paddles were picked up at the end of the week and transported to the laboratory in Ziploc® bags (SC Johnson, Racine, WI, USA), to evaluate the presence of eggs. The paddles with eggs were immersed in trays of water and were reared to adult stage.

Identification of sex and species was carried out with taxonomic keys [21]. Subsequently, the mosquitoes were placed in 1.5 ml conical tubes with 500 μl of DNA/RNA Shield™ (Zymo Research, Irvine, CA, USA) and stored at -80 °C.

### DNA isolation

The genomic DNA of the mosquitoes was isolated with a ZR Viral DNA/RNA Kit™ (Zymo Research) according to the manufacturer’s instructions. The quality and concentration of the isolated DNA were evaluated with a NanoDrop™ 2000c spectrophotometer (Thermo Scientific, Wilmington, DE, USA). Finally, samples were labeled and stored at -80 °C.

### PCR amplification

To identify the presence of *kdr* mutations in *Ae. aegypti* and *Ae. albopictus*, the previously proposed oligonucleotides AaSCF1, AaSCR4, AaSCF7 and AaSCR7 were used [22, 23]. These oligonucleotides allow the identification of five *kdr* sites within the VGSC sequence (Table 1).

**Table 1:** Oligonucleotides used for the identification of *kdr* mutations in DNA samples of *Ae. aegypti* and *Ae. albopictus* [22, 23]

Code	Sequence (5'-3')	Identified sites
AaSCF1	AGA CAA TGT GGA TCG CTT CC	Domain II, Segment 6
AaSCR4	GGA CGC AAT CTG GCT TGT TA	Ser989, Ile1011, Leu1014, Val1016
AaSCF7	GAG AAC TCG CCG ATG AAC TT	Domain III, Segment 6
AaSCR7	GAC GAC GAA ATC GAA CAG GT	Phe1534
AaSCF3	GTG GAA CTT CAC CGA CTT CA	Ser989, Ile1011, Leu1014,
AaSCF6	CGA CTT GAT CCA GTT GGA GA	Val1016
AaSCR8	TAG CTT TCA GCG GCT TCT TC	Phe1534

The mixture for the PCR was prepared with PCR Master Mix 2× (Promega, Madison, WI, USA) following the manufacturer’s specifications. The amplification process was carried out in a Mastercycler® gradient thermocycler (Eppendorf, Hamburg, Germany) based on a previously described protocol [22]. The quality and integrity of the PCR products were evaluated by agarose gel electrophoresis (AMRESCO, Solon, OH, USA), prepared at 1.5% and stained with Gel red® (Biotium, Fremont, CA, USA). Fragments of approximately 800 bp were obtained with the oligonucleotides AaSCF1 and AaSCR4. Fragments of approximately 700 bp were obtained with the oligonucleotides AaSCF7 and AaSCR7. The samples amplified by PCR were subsequently stored at -20 °C.

### Sequencing

The sequencing reactions were performed from the PCR products using the oligonucleotides AaSCF3, AaSCR6 and AaSCR8 [22, 23]. Sequencing was undertaken by Macrogen (Seoul, Korea), and the direct sequencing of the samples was performed on an ABI 3730XL genetic analyzer (Applied Biosystems, Foster City, CA, USA).

### Analysis of results

The chromatograms were visualized with BioEdit v.7.2.5 software [24]; the sequences obtained in ABI format was transformed to FASTA format for editing. The authenticity of the sequences was corroborated through the BLAST program (https://blast.ncbi.nlm.nih.gov/Blast.cgi), considering an identity percentage greater than 95%. The sequences were aligned and edited with MEGA 7.0 software [25]. To calculate the frequency of the *kdr* mutations, we considered the number of samples that showed point changes in the *kdr* sites of interest as a function of the total sequences analyzed.

## Results

A total of 3432 specimens of *Ae. aegypti* and 593 specimens of *Ae. albopictus* were collected. Of these, 149 specimens were sequenced, of which 74 individuals were *Ae. aegypti* and 75 were *Ae*. *albopictus*. From these, 447 sequences were obtained, of which only 194 were of sufficient quality to include in the analysis for detecting mutations at the level of Domain II of the VGSC protein. The *kdr* sites analyzed were Ser989, Ile1011, Leu1014, Val1016 and Phe1534, resulting in two point mutations (Ile1011Met and Val1016Gly) identified in a single specimen of *Ae. aegypti* from the locality of Nuevo Chorrillo. Table 2 shows the number of samples evaluated and positive samples in *Aedes* spp.

**Table 2:** *kdr* mutations detected in the samples of *Aedes* spp.

Domain	*kdr* sites	Evaluated sequences	Positive sequences
II	Ser989	78	1
	Ile1011		
	Leu1014		
	Val1016	96	1
III	Phe1534	20	0
Total		194	2

**Fig. 1:**
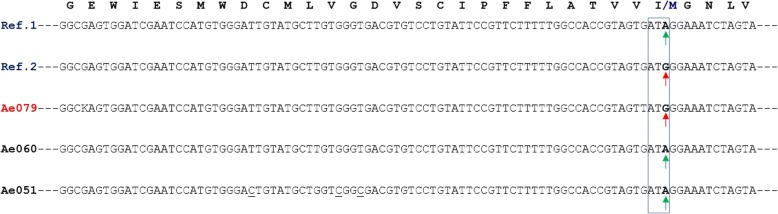
Partial section of the nucleotide sequence of DIIS6 of the VGSC protein (*para* gene) in *Ae. aegypti* (exon 20). Samples Ae079, Ae060 and Ae051 are contrasted with reference sequences Ref. 1 (GenBank: AB914690.1) and Ref. 2 (GenBank: FJ479612.1)

**Fig. 2:**
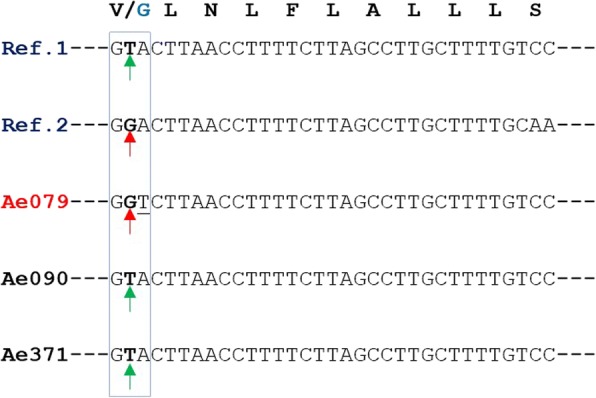
Partial section of the nucleotide sequence of DIIS6 of the VGSC protein (*para* gene) in *Ae. aegypti* (exon 21). Samples Ae079, Ae090 and Ae371 are contrasted with reference sequences Ref. 1 (GenBank: AB914690.1) and Ref. 2 (GenBank: AB914689.1)

The multiple alignments of the partial nucleotide sequence of the *para* gene in *Ae. aegypti* are presented, specifically from exons 20 and 21 in Figs. 1 and 2, respectively. These present the contrast of some of the sequences analyzed in the study with reference sequences obtained from the GenBank database.

In sample Ae079, the change in the amino acid isoleucine (Ile) by the amino acid methionine (Met) was observed, which is the product of a transition in the third base of the codon 1011; the non-mutated codon is ATA and the mutated codon is ATG (Additional files 1 and 2).

At position 1016 of sample Ae079, the change of the amino acid valine (Val) to the amino acid glycine (Gly) was observed, a product of a transversion occurring in the second nucleotide of the codon; the non-mutated codon is GTA and the mutated codon is GGT (Additional files 1 and 2). It is important to note that at the level of the third nucleotide there is also a point mutation (A → T), but it is synonymous.

The frequency of *kdr* mutations in the samples of *Ae*. *aegypti* evaluated in this study was 0.01, which corresponds to 1% of the total samples, and 0.06, which corresponds to 6% of the samples from Nuevo Chorrillo (Table 3).

**Table 3:** Samples of *Aedes* spp. with *kdr* mutations, according to the sampling location

Species	Location					Total
	Bethania	Garzas de Pacora	Nuevo Chorrillo	Princesa Mía	Lluvia de Oro	
*Aedes aegypti*	0/20	0/3	1/15 (0.06^a^)	0/5	0/17	1/60 (0.01^b^)
*Aedes albopictus*	0/2	0/2	0/64	0/0	0/3	0/71

^a^Frequency based on the samples of *Ae. aegypti* evaluated from Nuevo Chorrillo

^b^Frequency based on the total samples of *Ae. aegypti* evaluated in this study

## Discussion

Recent reports suggest that the populations of *Ae. aegypti* in Panama are, in general terms, susceptible to commercial pesticides. Larval resistance bioassays have shown the existence of vector populations that have moderate levels of resistance to the pyrethroid deltamethrin [8]. These results are significant given that, in the absence of adequate management by the vector control programme, the potential for the dissemination of this phenotypic trait may be increased in the future. However, the detection of point mutations Ile1011Met and Val1016Gly in a single specimen of *Ae. aegypti* using molecular tools demonstrates the importance and feasibility of incorporating molecular techniques for the detection and monitoring of resistance to insecticides in mosquitoes. This approach also complements the results of the bioassays and improves control strategies.

It is important to point out that the detection of mutation Val1016Gly raises new questions about its distribution and introduction at the regional level since, to our knowledge, it is the first time that is reported in America. In fact, the Val1016Ile and Val1016Gly mutations have distinct geographical distributions, with Val1016Gly found in Asia [26–30] and Val1016Ile in the Americas and recently detected in Africa [14, 26, 27, 31–34]. Based on these results, we hypothesize that the Val1016Gly mutation could have been introduced to Panama *via* the transit of containers or tires with eggs because the country is a site of commercial and international transit, which is difficult to monitor and control. At first, we hypothesized that the mutation was introduced in America but there is also the possibility that it is a new mutation. If it is a new mutation, an exhaustive phylogeographical analysis will establish the origin of the mutation present in our region.

In the present study, both the Ile1011Met and Val1016Gly mutations were detected in a sample of *Ae. aegypti* from the same locality (NC). The co-occurrence of *kdr* mutations in *Ae. aegypti* has been reported previously, specifically of the Val1016 and Ser989 mutations in Asian populations of this vector [26, 27]. However, the implications of the combination of *kdr* mutations are debated; some studies report that their co-occurrence improves resistance [35], but others conclude that there is no additive or synergistic effect [36].

In this study it was not possible to detect *kdr* mutations in *Ae. albopictus* sequences, a fact that may be related to technical factors (due to the quality of some sequences obtained) which we consider part of the limitations of this study. It is important to note that of the 194 evaluated sequences, only 20 were found to have sufficient quality to identify point changes at the DIIIS6 level of the VGSC, a region where it has been possible to characterize the Phe1534Cys mutation. Notably, a study conducted in Costa Rica [22] did not detect mutant alleles associated with *kdr* resistance in a natural population of *Ae. albopictus*, a characteristic attributed to the recent invasion of this species in that country. The colonization of *Ae. albopictus* populations in Panama is relatively recent [37] compared to *Ae. aegypti* [38]; therefore, the failure to detect *kdr* mutations in *Ae. albopictus* may be because chemical control with insecticides has not yet put pressure on the populations of this vector. However, in *Ae. albopictus* populations from other latitudes, it has been possible to detect the Phe1534Cys mutation. For example, in Singapore, Kasai et al. [39] found that 92.3% of mosquitoes exhibited the Phe1534Cys mutation as detected by sequencing, thus estimating a frequency of 73.1% for the Cys1534 allele. Recently, the evaluation of *Ae. albopictus* populations from Asia, Africa, America and Europe [40] detected two new *kdr* mutations at the level of domain III of the VGSC, namely mutations Ile1532Thr and Phe1534Ser, the latter presenting a significant association with resistance to deltamethrin.

The frequency of *kdr* mutations in the analyzed samples was low (1%) compared to that reported in studies conducted in the region. For example, in populations of *Ae. aegypti* from Grand Cayman and Cuba, frequencies of 79% and 51%, respectively, for the *kdr* allele Ile1016 were detected through sequencing [41, 42]. Similar results are reported for populations of *Ae. aegypti* from Venezuela evaluated through the allele-specific PCR technique (PCR-AS) [43] and from Brazil [44]. Another study carried out in Brazil [31] revealed patterns of regional distribution of *kdr* mutations attributed to positions Val1016 and Phe1534 in *Ae. aegypti* collected over ten years. According to the authors, the regionalization of the *kdr* alleles reflects differences in the populations of *Ae. aegypti* that colonized the continent.

Lastly, we consider that the low frequency detected in our study does not yet have an impact on mosquito control interventions. Determining the distribution of the Ile1011Met and Val1016Gly mutations, as well as other *kdr* mutations in populations of *Ae. aegypti* or in other species of mosquito vectors present in Panama, requires a greater sampling effort and an adaptation of the methodology used in this research. The information generated will be of great value in determining the frequency of the mutant alleles.

## Conclusions

This study provides evidence for a low frequency of *kdr* mutations (Ile1011Met and Val1016Gly) in *Ae. aegypti* populations in Panama. The low frequency recorded is perhaps not enough to have an impact on the interventions of mosquito control. To our knowledge, we report, for the first time in America the Val1016Gly mutation documented in Asia. The finding of the *kdr* mutations in specimens not previously exposed to resistance bioassays is indicative that the natural populations of this vector could be developing resistance to the insecticides that are being applied in Panama. In general terms, the information on the presence of this *kdr* mutation in Panama can help monitor the spread of the mutation in America in the case that it becomes a significant problem for vector control. This result is highly relevant to the *Aedes* Control Programme in Panama since it constitutes a feasible approach for the timely detection of resistance as well as for the development of strategies.

## Additional files

Additional file 1: Alignment of the newly generated sequences. Partial section of the nucleotide sequence of DIIS6 of the VGSC protein (*para* gene) in *Ae. aegypti* (exon 20). (FAS 0.608)

Additional file 2: Alignment of the newly generated sequences. Partial section of the nucleotide sequence of the DIIS6 of the VGSC protein (*para* gene) in *Ae. aegypti* (exon 21). (FAS 0.243)

## Abbreviations

A: Adenine
C: Cytosine
CHIKV: Chikungunya virus
DENV: Dengue virus
G: Guanine
*kdr*: Knockdown resistance
MINSA: Ministerio de Salud (Ministry of Health)
PCR: Polymerase chain reaction
PCR-AS: Polymerase chain reaction allele-specific
T: Thymine
VGSC: Voltage-gated sodium channel
YFV: Yellow fever virus
ZIKV: Zika virus
